# A Case of Difficult Arterial Cannulation: Is Intra-Arterial Blood Pressure Monitoring an Absolute Requirement for Paediatric Liver Transplantation?

**DOI:** 10.3390/jcm12134387

**Published:** 2023-06-29

**Authors:** Helen McKevitt, Zoka Milan

**Affiliations:** Department of Anaesthesia, Kings College Hospital, London SE5 9RS, UK

**Keywords:** liver transplant, monitoring, arterial blood pressure, haemodynamic, paediatric

## Abstract

Invasive arterial blood pressure monitoring is the standard of practice in terms of intraoperative blood pressure surveillance during liver transplantation. While this is an ideal, achieving reliable arterial access can be extremely challenging in the paediatric and neonatal population, repeated attempts at arterial cannulation are not without risk and alternative best practice means of haemodynamic monitoring are not clearly established. We describe a case of paediatric liver transplantation in a 3.9 kg infant that was complicated by difficult arterial cannulation, and we suggest that, when reasonable attempts to achieve intra-arterial access have failed, it is safe to proceed with paediatric liver transplantation with non-invasive blood pressure monitoring at 2 min intervals throughout the case and 1 min intervals at reperfusion. We recognise the unique technical challenges in paediatric liver transplant anaesthesia, and we advocate for the establishment of formal clinical training competencies in line with adult practice recommendations. We recommend the use of the Seldinger technique under ultrasound guidance as a first-line approach when difficult arterial cannulation is anticipated, and we discuss techniques for alternative approaches. We suggest that additional alternative means of haemodynamic monitoring should be considered when arterial access cannot be established; however, as no method demonstrates absolute superiority, one or a combination of techniques should be considered, depending on local availability and expertise.

## 1. Introduction

Intra-arterial monitoring is the mainstay of blood pressure surveillance during liver transplantation (LT). The reason for this is multifactorial—LT involves major open abdominal cavity surgery, there is potential for massive blood loss and there is an expectation of haemodynamic instability related to manipulation of the vena cava and reperfusion of the donor organ.

Children, however, tend to be more haemodynamically stable than adults, and achieving reliable arterial access can be extremely technically challenging due to the small vessel diameter, particularly in the neonatal population. Difficult arterial cannulation is further exacerbated in children who have had prolonged paediatric intensive care unit (PICU) admissions, as they often have had multiple previous attempts at arterial cannulation and may be receiving treatment with vasoconstrictors, which can contribute to vasospasm. There may be significant risks involved with repeated arterial cannulation, including vessel thrombosis, limb ischemia and, rarely, loss of the limb. Additionally, there may be greater time pressure and a higher proportion of night time work with limited access to expert assistance due to the acuity of many paediatric presentations. With these issues in mind, the practicalities and true benefit of exhaustive attempts at arterial cannulation should be taken in context with the risk of potential harm to the patient and excessive delays to the procedure.

While we anticipate that the issue of failed or impossible intra-arterial access is perhaps not uncommon in paediatric LT practice, we were unable to source any recommendations in the literature about how to proceed in terms of best practice. We present a case of a paediatric LT that proceeded successfully with non-invasive blood pressure (NIBP) monitoring and suggest this as a reasonable approach when proportionate attempts to obtain intra-arterial access have failed. We used NIBP monitoring on the upper arm, which we cycled at 2 min intervals throughout the case and at 1 min intervals at reperfusion, plus a standard optimisation of fluid balance, haemoglobin, temperature and potassium levels.

As a large tertiary centre providing both adult and paediatric transplant services, we particularly recognise the technical challenges in caring for small children, and we suggest that recommended competencies for transplant anaesthesia in children should be formalized—there are clear recommendations for competencies in adult practice; however, this is not yet the case in paediatrics [1].

We discuss the role of blood pressure monitoring in children and compare IABP with NIBP. We also discuss the approach to a child with difficult arterial access in terms of alternative techniques for obtaining intra-arterial access and alternative means of the monitoring of cardiac output and/or tissue perfusion intraoperatively—these are well established techniques in other areas of paediatric anaesthetic practice; however, they are less well described in paediatric LT [2].

## 2. Case Report

The case we present is of a 3.9 kg 3-month-old child with acute liver failure (ALF) secondary to thyrosinaemia, listed for super urgent LT following prolonged hospitalisation and PICU admission. The child was born at term and had initially been well; however, at 2 months of life she was admitted to the hospital with a suspected bowel obstruction and underwent an exploratory laparotomy. During this admission she was found to be coagulopathic with significant metabolic derangement—INR (international normalised ratio) 5.1, hypoglycaemia, bilirubin 70 umol/L, ammonia 136 umol/L. Transfusion requirements during the laparotomy were significant—red cells 20 mL/kg, fresh frozen plasma (FFP) 10 mL/kg and cryoprecipitate 10 mL/kg. A subsequent diagnosis of ALF secondary to thyrosinaemia was made and the child was listed for super urgent LT. She was managed in PICU over a period of 4 weeks while awaiting a suitably sized graft, where she received multiorgan support including mechanical ventilation, haemodynamic support and renal replacement therapy. Throughout this time, she had considerable ongoing transfusion requirements including six hourly FFP and cyroprecipitate plus daily requirements for red cell and platelet transfusions. She had multiple invasive lines during her PICU stay, both venous and arterial.

At 3 months of life, a suitable donor was identified; however, the donor graft was not ideal. This was a donation after brainstem death (DBD) donor, who was young but had lifestyle factors that were high risk. The cold ischaemia time was long at 14 h and the split graft weight was three times larger than the recipient’s liver weight, posing potential surgical challenges and risk of greater than average haemodynamic compromise at reperfusion.

The on-call team were due to start the case in the early evening; however, they did not begin until night time due to unforeseen delays. At preoperative review in PICU, the child was intubated and ventilated with moderate respiratory support and moderate cardiovascular support with noradrenaline 0.1 mcg/kg/min. She was anuric, on continuous veno-veno haemofiltration and, of note, had widespread tissue oedema. In terms of access, there was established central venous access in the left femoral vein, a vascath in the left internal jugular and an arterial line in the left radial artery. Significantly, there was a haematoma in the right femoral region from a previous biopsy at this site. In the context of the ongoing coagulopathy, lines were noted to be oozy. At some stage during the transfer to the theatre, the radial arterial line was unfortunately lost.

With the child anaesthetised on the operating table, there were multiple failed attempts to secure a new arterial line. Initial attempts were made using a simple 22-gauge Abbocath cannula guided by the palpation of pulses under direct vision. With this technique, the right radial artery was successfully punctured by the consultant anaesthetist but it was not possible to thread the cannula along the vessel. The brachial artery was identified as the next best option, and attempts were made on both the right and left brachial arteries using a similar technique, which again failed. An adjustment in technique was made and there were further attempts with a 22-gauge Vygon cannula using the Seldinger technique with the addition of ultrasound guidance. Cannulation of the right femoral artery and both axillary arteries were attempted with this technique; however, again, the artery could be punctured with the introducer needle but it was not possible to pass the guidewire. Attempts at femoral cannulation were made more challenging by a recent biopsy taken from the left groin. In addition, the patient was on a moderate dose of noradrenaline (0.1 mg/kg/min), which may have contributed to vasoconstriction and vasospasm.

At this stage, the consultant paediatric intensivist and senior anaesthetic registrar were called to assist attempts; however, they were also unsuccessful. Given that the case was being done overnight, there were limited options for calling on further skilled practitioners. After 1.5 h, a suggestion was made by the surgeon to insert an arterial cannula directly into the abdominal aorta; however, as the patient was felt to be relatively stable following a risk–benefit discussion, this idea was abandoned. The decision was made to proceed with the transplant with NIBP monitoring.

Anaesthesia was maintained with isoflurane and nitrous oxide plus infusions of fentanyl, atracurium and noradrenaline. Standard monitoring with oxygen saturations (SO_2_), electrocardiography (ECG), end-tidal carbon dioxide (ETCO_2_), temperature and central venous pressure (CVP) were in place. NIBP measurements at the upper arm were recorded every 2 min during the majority of the case and every 1 min at reperfusion. Median systolic pressures were maintained between 75–80 mmHg and median diastolic pressures were between 35–40 mmHg. Regular blood gas measurements were taken from the CVC in order to guide haemoglobin and potassium management. Intraoperative transfusion requirements were guided by thromboelastography (TEG) and included FFP, cryoprecipitate—60 mL/kg, platelets—50 mL/kg, packed red cells—60 mL/kg plus tranexamic acid—20 mg/kg and the additional infusion of crystalloid with plasmalyte—40 mL/kg.

The child had a reduced segment 2 and partial segment 3 LT via the piggyback technique in order to preserve the recipient venous return through the inferior vena cava (IVC). The abdomen was left open due to the relatively large size of the donor segment. The child was stable throughout the procedure and had good graft reperfusion with a small amount of urine production intraoperatively.

The child was managed post operatively in PICU without IABP monitoring and inotropes were weaned on day 1 post operatively. A lump was noted in the left brachial artery, which was noted to be reducing in size—there were no issues with perfusion of the limb.

## 3. Discussion

### 3.1. Training and Competencies

At King’s, we perform approximately 200 adult and 40 paediatric LTs per year [3]. We are one of the largest paediatric LT centres in the UK and we are one of a small number of centres that provide both adult and paediatric transplant services, including transplants for small babies and neonates—further information on UK transplant practices can be found through. At our centre, both adult and paediatric LT services are provided by the same anaesthetic team, led by eight dedicated LT consultant anaesthetists. At King’s, we know that we have relatively large volume transfusion requirements and, traditionally, our consultant appointments have reflected this issue, favouring those with expertise in haemodynamic management rather than those with extensive formal paediatric experience [4]. We maintain that this expertise in haemodynamic management remains vital given our large transfusion requirements, and that it is probably of greater importance than paediatric skills per se. However, we do increasingly recognise the benefits of formal paediatric training in addition to haemodynamic management skills; recent consultant appointments are reflective of this, specifying a requirement of 12 months of formal advanced paediatric training in addition to 12 months of dedicated LT anaesthesia. While our centre has elected to implement these training requirements, there are no clear national or international recommendations for clinical competencies specific to paediatric LT services.

Recommendations for adult transplant team anaesthetic competencies have recently been published by the Society for the Advancement of Transplant Anaesthesia (SATA) in North America [1]. This document makes an extensive range of recommendations in terms of clinical competencies, including: pre-operative assessment, technical skills, medical knowledge, professionalism and communication. These recommendations are presented in clear, easy to follow practice-based learning milestones targeted towards fellows in transplant anaesthesia. A similar document relating to paediatric practice would be of particular use in a department such as our own, which provides both adult and paediatric services and has a well-established fellowship programme.

### 3.2. Technique and Alternative Approaches

In our case, initial efforts to gain intra-arterial access were made using a simple ‘Abbocath’ cannula using anatomical landmarks before moving on to the Seldinger (guidewire) technique using ultrasound guidance. There is now good evidence to suggest that the Seldinger technique has an improved first-time success rate in radial arterial cannulation in the neonatal population [5] and that the use of ultrasound improves initial success rate [6]. As ready access to high quality ultrasound machines is ever more available and clinical experience with ultrasound is increasing, we strongly suggest that the Seldinger technique under ultrasound guidance should be considered as the first technique of choice in the neonatal and paediatric populations, particularly when there is anticipated difficulty. The use of a ‘Babywire’ guidewire, which is compatible with a 24-gauge needle or 2.0 French catheter may also be beneficial.

The surgical cut down approach has long been established as an acceptable technique for difficult arterial access in various cardiac catheterisation procedures in the paediatric population; for example, the surgical cut down of the carotid artery is not uncommonly used for access in the treatment of specific types of cardiac lesions. The femoral arterial cut down as another option is a well-established technique for transcatheter aortic valve implantation (TAVI) in adults, but is less well described in children [7]. While it remains a potential option, the surgical cut down approach requires paediatric surgical expertise that may or may not be available depending on local experience, plus potential vascular surgical support in the event of a complication, which, again, may not be available. The surgical cut down approach is also not without risk. In a case control study, Viswanathan et al. found that there were significant short term sequalae associated with the surgical cut down of the axillary artery in children under 2 years of age, with 23% of patients requiring heparin infusions for limb ischemia; however, there was no clear evidence of long-term harm [8].

In our case, the surgical consultant suggested aortic cannulation as an alternative technique for obtaining IABP monitoring. This is not an unfamiliar technique as our surgeons are occasionally required to obtain access for cardiopulmonary bypass; however, there is limited precedence for this technique when used for the indication of monitoring alone.

In a single centre case series, Polat suggests the use of a carotid artery cannulation via the percutaneous approach as safe and effective means of obtaining access for certain cardiac catheterisation procedures in infants and even very small preterm babies [9]. In our case, attempts were made in the radial, brachial, femoral and axillary arteries, while the carotid approach was not considered. As anaesthetists, we are familiar with the percutaneous approach and are accustomed to visualising the ultrasonographic anatomy of the neck. Percutaneous cannulation of the carotid artery may be a reasonable, practical and safe approach, particularly where surgical expertise and support is lacking.

### 3.3. Blood Pressure Monitoring in Children

What we are really interested in with any haemodynamic monitoring tool is tissue oxygenation. Transport of oxygen is heavily influenced by cardiac output (CO) or flow, which can be difficult to measure directly. Blood pressure (BP) measurements are clinically useful indicators of flow, as BP has a direct relationship with CO and is easily measurable [10].

CO = BP × SVR

While by no means a perfect surrogate marker, BP is a useful tool to approximate CO, particularly when interpreted with other indicators of flow such as ETCO_2_, capillary refill time, urine output and other techniques such as cerebral near infra-red spectroscopy (NIRS), which will be discussed.

While IABP is undoubtedly the gold standard, NIBP can still provide reliable, accurate information when used appropriately. Studies suggest that under general anaesthesia, systolic blood pressures measured via NIBP tend to read higher than IABP [11]. The degree of variation can be considerable, but is within 10 mmHg in the majority of cases—this should be taken into account when interpreting measurements [12]. The evidence suggests that this discrepancy between NIBP and IABP is minimised when BP cuffs are placed on the upper arm when compared to other sites. In a small prospective study of children aged 0–8 undergoing general anaesthesia, Hayes et al. found that differences in systolic NIBP and IABP of >10 mmHg were found in 20% of measurements recorded on the upper arm, compared to 27% of measurements recorded on the leg [12]. The importance of using appropriately sized BP cuffs in children cannot be over-emphasised, as a cuff that is too small will provide falsely high readings and a cuff that is too large will provide falsely low readings.

The use of IABP provides continuous, real-time measurements of arterial blood pressure. This is clearly not achievable with NIBP, which raises the question of how frequently NIBP measurements should take place. There are no clear recommendations in the literature regarding the frequency of NIBP measurements in paediatric LT or other comparable major surgical procedures. We suggest that when IABP monitoring is not achievable, NIBP measurements should be taken every 2 min throughout the case and increased to every 1 min during reperfusion. We feel that this is a reasonable approach, as children tend to have a greater degree of haemodynamic stability than adults during LT surgery. Underlying differences in physiology such as a greater contribution of heart rate towards CO, and little/no underlying cardiovascular disease, e.g., vascular stenosis, which means that children tend to compensate well with the haemodynamic shifts that occur during LT.

### 3.4. Alternative Means of Monitoring Cardiac Output and/or Tissue Perfusion

In the vast majority of LTs, intraoperative monitoring techniques include IABP and CVP, with or without other non-invasive, minimally invasive or invasive monitors of tissue perfusion and/or CO. Traditionally, the specific monitoring of CO and/or tissue perfusion have not been in regular use in our paediatric LT practice at King’s; however, we recognise that these techniques should be considered as a supplemental means of monitoring, particularly when arterial access has not been achievable. There are various options without the overwhelming evidence of superiority of any one technique; therefore, it may be prudent to use one or indeed a combination of techniques, depending on local experience and availability.

Near-infrared spectroscopy (NIRS) is a non-invasive technique that is increasingly used for the intraoperative monitoring of cerebral perfusion (CP). NIRS utilizes the Beer-Lambert Law, which states that there is a linear relationship between the concentration of a solution and the absorption of light of a given wavelength passing through that solution [13]. Clinically, this is used to measure the oxygen saturation of haemoglobin by utilising probes placed on the forehead, which transmit infrared light that passes through the tissues and measures absorption of light in the range for oxy- and deoxyhemoglobin. This gives an idea of regional perfusion in the underlying tissue, which is extrapolated and used as a surrogate for underlying CP. The clear advantage of NIRS is that it is a non-invasive technique, it is simple to use and the probe is unlikely to become detached intraoperatively. Advocates of the technique also suggest that NIRS may more accurately represent CP when compared with central haemodynamic measurements, e.g., IABP, given that CP is relatively independent of central perfusion due to cerebral autoregulation [14]. Mainly, the evidence for this technique is in paediatric cardiac surgery, where there is good evidence to suggest that a combination of NIRS and transcranial doppler improves neurological outcome [15]. There is also a clinical correlation between NIRS and jugular venous oximetry, which, while not the gold standard, is often considered a standard for the comparison of other techniques [14].

Of note, in their systematic review, Ghidini et al. suggest that NIRS overlying the skin is beneficial in the monitoring of graft perfusion and in particular, in reducing the risk of hepatic artery thrombosis (HAT) in paediatric kidney and liver transplantation [16]. However, this is a novel technique for this specific indication and the authors conclude that further investigation is required to establish the normal range of NIRS values and the factors affecting NIRS monitoring.

The transcranial doppler (TCD) is based on the principle of the Doppler effect, whereby ultrasound waves emitted from the doppler probe are reflected by moving red cells within the intracerebral vasculature. The difference between the frequency of the emitted and reflected waves is proportional to the velocity of blood flow, assuming the diameter of the vessel remains constant. TCD involves placing a doppler probe on the temporal bone in order to measure the velocity of flow within the middle cerebral artery [17]. While TCD is a non-invasive technique, there are a number of technical challenges that may reduce accuracy—inconsistent doppler signalling, loss of probe contact, incorrect angulation of the probe and inadequate anatomical window for ultrasonic interrogation—a particular issue in small children [17]. Similarly to NIRS, the main evidence in support of TCD is in paediatric cardiac surgery, where an improved neurological outcome is suggested.

Continuous central venous oxygen saturation (ScvO_2_) is a well-established means of the intraoperative monitoring of tissue oxygenation, again with predominant evidence in cardiac surgery, where it is comparable to NIRS [13]. This, of course, requires central venous cannulation and is therefore invasive; however, central venous cannulation is a standard of practice in LT, so an additional procedure would not be required. ScvO_2_ is measured continuously via a fibreoptic catheter within the central venous line; this utilises reflection spectrophotometry to measure the oxygen content of blood returning to the right side of the heart. When the oxygen supply is insufficient to meet the metabolic demands of the tissues, this results in an abnormal SvO_2_, which is reflective of inadequate systemic oxygenation [18].

In terms of intraoperative CO monitoring, the gold-standard is the transoesophageal echo (TOE). It is minimally invasive and provides a real time visualisation of ventricular function and haemodynamic volume status [19]. While TOE has become widespread in adult LT practice [20], there is less extensive documentation of its use in paediatrics. This is likely to be related to a number of factors: poor availability of suitable equipment, issues with expertise and training and a feeling of less clinical usefulness in paediatrics, given the superior ability to compensate for hemodynamic changes. With these considerations, it remains to be seen whether the use of TOE in paediatrics will increase in line with adult practice.

Non-invasive CO monitoring via thoracic bioimpedance is a potential future development that may be useful in paediatric LT. However, as yet, there is limited, mixed evidence in other areas of paediatric surgical practice [20,21], and its useful in LT is not fully validated [22].

## 4. Conclusions

While IABP monitoring remains a mainstay of haemodynamic monitoring for paediatric LT, this can be extremely technically challenging in small children and infants. When reasonable attempts to achieve intra-arterial access have failed, our experience suggests that it is safe to proceed with paediatric LT with NIBP monitoring every 2 min (increased to 1 min at reperfusion), in addition to other standards of care.

When difficult arterial cannulation is anticipated, the use of the Seldinger technique with ultrasound guidance is recommended as the first-line technique of choice. Alternative anatomical sites for intra-arterial cannulation can be considered for both the percutaneous and/or surgical approach; however, this should be performed in accordance with the local experience and expertise, and a risk vs. benefit discussion should take place. While as yet unvalidated in paediatric LT, alternative means of haemodynamic monitoring such as NIRS, TCD and ScvO_2_ are well validated in other areas of paediatric anaesthetic practice. If IABP monitoring cannot be achieved, then alternative single or multimodal monitoring should be strongly considered—as no single method demonstrates absolute superiority, the technique/s of choice should be led by local availability and experience. NIRS and/or TCD may also serve as a useful adjunct to IABP monitoring as a standard of practice in paediatric LT, as these techniques potentially provide a more accurate picture of cerebral oxygenation than IABP.

Finally, we advocate for the establishment of formal clinical competencies for training in paediatric transplant anaesthesia in line with the recently published adult recommendations, and suggest that this may improve success in cases of challenging arterial cannulation.

## Data Availability

Data sharing not applicable.

## References

[1] Nguyen-Buckley C., Wray C.L., Zerillo J., Gilliland S., Aniskevich S., Nicolau-Raducu R., Planinsic R., Srinivas C., Pretto J.E.A., Mandell M.S. (2019). Recommendations From the Society for the Advancement of Transplant Anesthesiology: Liver Transplant Anesthesiology Fellowship Core Competencies and Milestones. Semin. Cardiothorac. Vasc. Anesth..

[2] Abdul-Khaliq H., Troitzsch D., Berger F., Lange P.E. (2000). Regional transcranial oximetry with near infrared spectroscopy (NIRS) in comparison with measuring oxygen saturation in the jugular bulb in infants and children for monitoring cerebral oxygenation. Biomed. Tech. Biomed. Eng..

[3] NHS Blood and Transplant Annual Report on Liver Transplantation. September 2022..

[4] Milan Z., Katyayani K., Cubas G., Unic-Stojanovic D., Cooper M., Bras P., Macmillan J. (2019). Trends in transfusion practice over 20 years in paediatric liver transplant programme. Vox Sang..

[5] Jang Y.E., Kim E.H., Lee J.H., Kim H.S., Kim J.T. (2019). Guidewire-assisted vs. direct radial arterial cannulation in neonates and infants: A randomised controlled trial. Eur. J. Anaesthesiol..

[6] Schindler E., Schears G.J., Hall S.R., Yamamoto T. (2012). Ultrasound for vascular access in pediatric patients. Pediatr. Anesth..

[7] Abdelaziz H.K., Megaly M., Debski M., Rahbi H., Kamal D., Saad M., Wiper A., More R., Roberts D.H. (2020). Meta-Analysis Comparing Percutaneous to Surgical Access in Trans-Femoral Transcatheter Aortic Valve Implantation. Am. J. Cardiol..

[8] Viswanathan S., Arthur R., Evans J., Truscott J., Thomson J., Gibbs J. (2012). The early and mid-term fate of the axillary artery following axillary artery cut-down and cardiac catheterization in infants and young children. Catheter. Cardiovasc. Interv..

[9] Polat T.B. (2020). Use of percutaneous carotid artery access for performing pediatric cardiac interventions: Single-center study. Ann. Pediatr. Cardiol..

[10] Karlsson J., Lönnqvist P. (2021). Blood pressure and flow in pediatric anesthesia: An educational review. Pediatr. Anesth..

[11] Wax D.B., Lin H.-M., Leibowitz A.B. (2011). Invasive and Concomitant Noninvasive Intraoperative Blood Pressure Monitoring: Observed Differences in Measurements and Associated Therapeutic Interventions. Anesthesiology.

[12] Hayes S., Miller R., Patel A., Tumin D., Walia H., Hakim M., Syed F., Tobias J.D. (2019). Comparison of blood pressure measurements in the upper and lower extremities versus arterial blood pressure readings in children under general anesthesia. Med. Devices Évid. Res..

[13] Rao A., Gourkanti B., Van Helmond N. (2021). Near-Infrared Spectroscopy Monitoring in Pediatric Anesthesiology: A Pro-Con Discussion. Cureus.

[14] Kakuta N., Kawahito S., Mita N., Kambe N., Kasai A., Wakamatsu N., Katayama T., Soga T., Tada F., Kitaichi T. (2013). Usefulness of central venous oxygen saturation monitoring during bidirectional Glenn shunt. J. Med. Investig..

[15] Polito A., Ricci Z., Di Chiara L., Giorni C., Iacoella C., Sanders S.P., Picardo S. (2006). Cerebral blood flow during cardiopulmonary bypass in pediatric cardiac surgery: The role of transcranial Doppler—A systematic review of the literature. Cardiovasc. Ultrasound.

[16] Ghidini F., Benetti E., Zucchetta P., Amigoni A., Gamba P., Castagnetti M. (2021). Transcutaneous near-infrared spectroscopy (NIRS) for monitoring kidney and liver allograft perfusion. Int. J. Clin. Pract..

[17] Mittnacht A.C., Rodriguez-Diaz C. (2014). Multimodal neuromonitoring in pediatric cardiac anesthesia. Ann. Card. Anaesth..

[18] Chetana Shanmukhappa S., Lokeshwaran S. (2021). Venous Oxygen Saturation.

[19] Vetrugno L., Barbariol F., Baccarani U., Forfori F., Volpicelli G., Della Rocca G. (2017). Transesophageal echocardiography in orthotopic liver transplantation: A comprehensive intraoperative monitoring tool. Crit. Ultrasound J..

[20] Soong W., Sherwani S.S., Ault M.L., Baudo A.M., Herborn J.C., De Wolf A.M. (2014). United States Practice Patterns in the Use of Transesophageal Echocardiography During Adult Liver Transplantation. J. Cardiothorac. Vasc. Anesth..

[21] Tomaske M., Knirsch W., Kretschmar O., Woitzek K., Balmer C., Schmitz A., Bauersfeld U., Weiss M., Working Group on Non-invasive Haemodynamic Monitoring in Paediatrics (2008). Cardiac output measurement in children: Comparison of Aesculon cardiac output monitor and thermodilution. Br. J. Anaesth..

[22] Wang D.-J., Lee I.-S., Chou A.-H., Chen C.-Y., Ting P.-C., Teng Y.-H., Lin J.-R., Tsai H.-I. (2018). Non-invasive cardiac output measurement with electrical velocimetry in patients undergoing liver transplantation: Comparison of an invasive method with pulmonary thermodilution. BMC Anesthesiol..

